# Supplementary material for: Wax worm saliva and the enzymes therein are the key to polyethylene degradation by Galleria mellonella
Source: Nat Commun. 2022 Oct 4;13:5568. doi: 10.1038/s41467-022-33127-w (PMC9532405; doi:10.1038/s41467-022-33127-w)
Supplement: Supplementary file 1 — Supplementary Information [file 41467_2022_33127_MOESM1_ESM.pdf]

Supplementary Materials for

Title:
Wax worm saliva and the enzymes therein are the key to polyethylene degradation by *Galleria mellonella*

A. Sanluis-Verdes[1§], P. Colomer-Vidal[1§], F. Rodriguez-Ventura[1], M. Bello-Villarino[1], M. Spinola-Amilibia [2], E. Ruiz-Lopez[3], R. Illanes-Vicioso[3], P. Castroviejo[4], R. Aiese Cigliano[5], M. Montoya[6], P. Falabella[7], C. Pesquera[8], L. Gonzalez-Legarreta[8], E. Arias-Palomo[2], M. Solà,[3] T. Torroba[4], C.F. Arias[1*] and F. Bertocchini[1*]

Correspondence to: federica.bertocchini@csic.es, tifar@ucm.es

Supplementary Figures

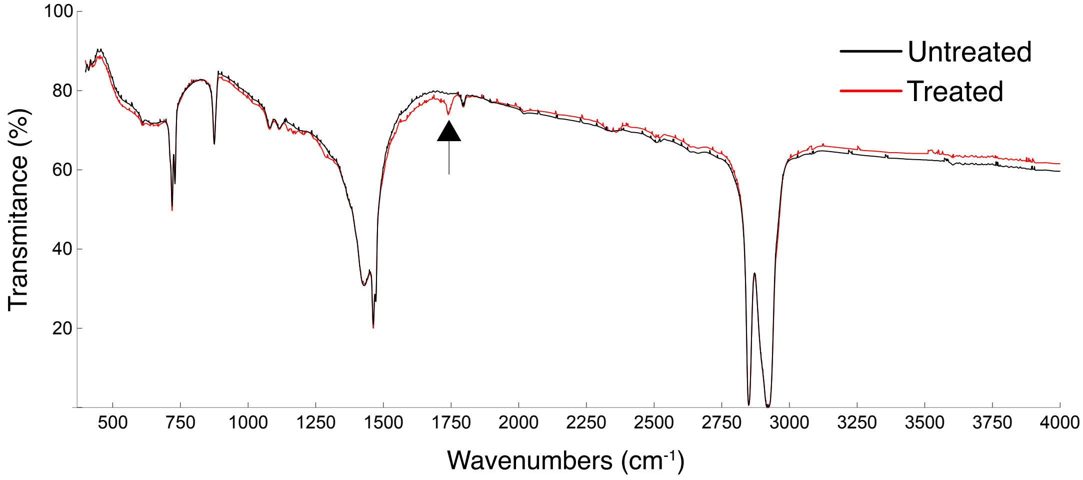

Supplementary Fig. 1. FTIR of PE film treated with GmSal. The arrow indicates the peak at around 1750 cm^{-1} (i.e. oxidation-carbonyl group). Source data are provided as a Source Data file.

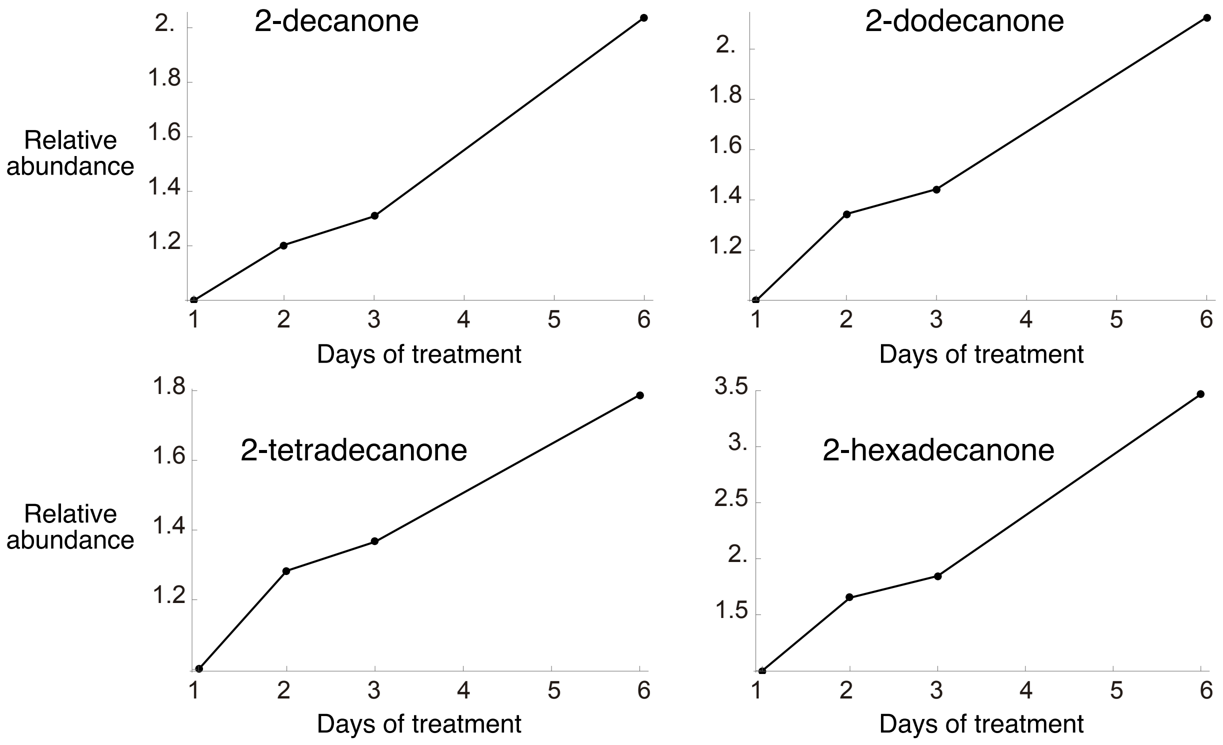

Supplementary Fig. 2. Relative abundance of the ketones 2-decanone, 2-dodecanone, 2-tetradecanone, 2-hexadecanone measured at day 1, 2, 3 and 6 of PE treatment with GmSal. Source data are provided as a Source Data file.

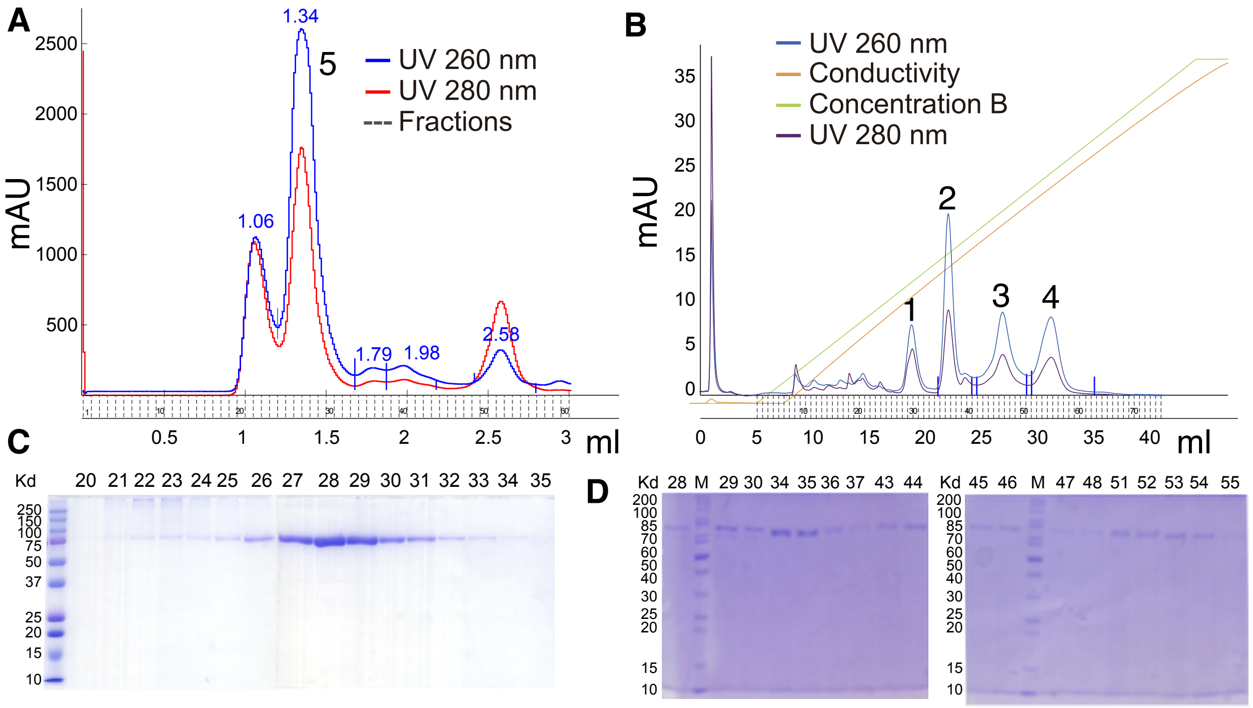

Supplementary Fig. 3. Chromatographic analyses of saliva samples. A, B. Size exclusion chromatography (A), and ion exchange chromatography (B). C. SDS-gel of the fractions in A. D. SDS-gel of the fractions in B. Experiments in figure C and D were performed twice. Source data are provided as a Source Data file.

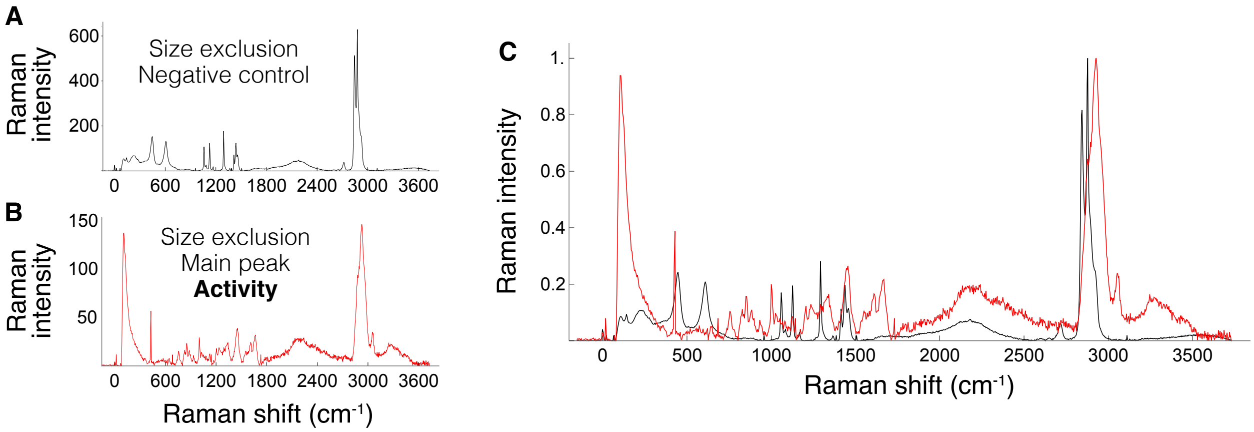

Supplementary Fig. 4. Functional analysis of degradation activity in the size exclusion main fraction (peak 5 in Fig. S3A). A, B AFM-RAMAN spectroscopy of control (A) and peak 5-treated PE film (B). A. Control PE film (see Fig. 1 for details). B. Punctual analyses of treated PE film, indicating PE deterioration. The spectrum shows an intense peak below 600 cm^{-1}, indicating an increase in additive detection; peaks at 711 and 1747 cm^{-1} correspond to the C=O group; oxidation also indicated between 3000-3500 cm^{-1}, and PE deterioration revealed by the broad peak between 1500 and 2400 cm^{-1}. C. Overlapping of A and B. Source data are provided as a Source Data file.

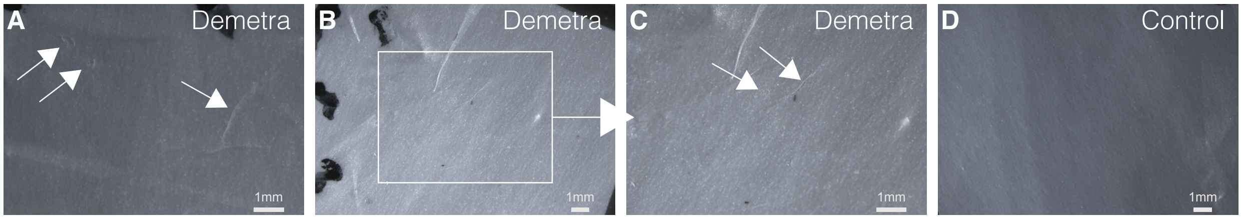

Supplementary Fig 5. Demetra effect on PE film. A-D. Demetra causes diverse extent of damage after application on PE film, with some visible external signs, (A and magnification in B) to milder, barely visible effects (C, arrows). D. Control. Experiments in this figure were performed multiple times (>5).

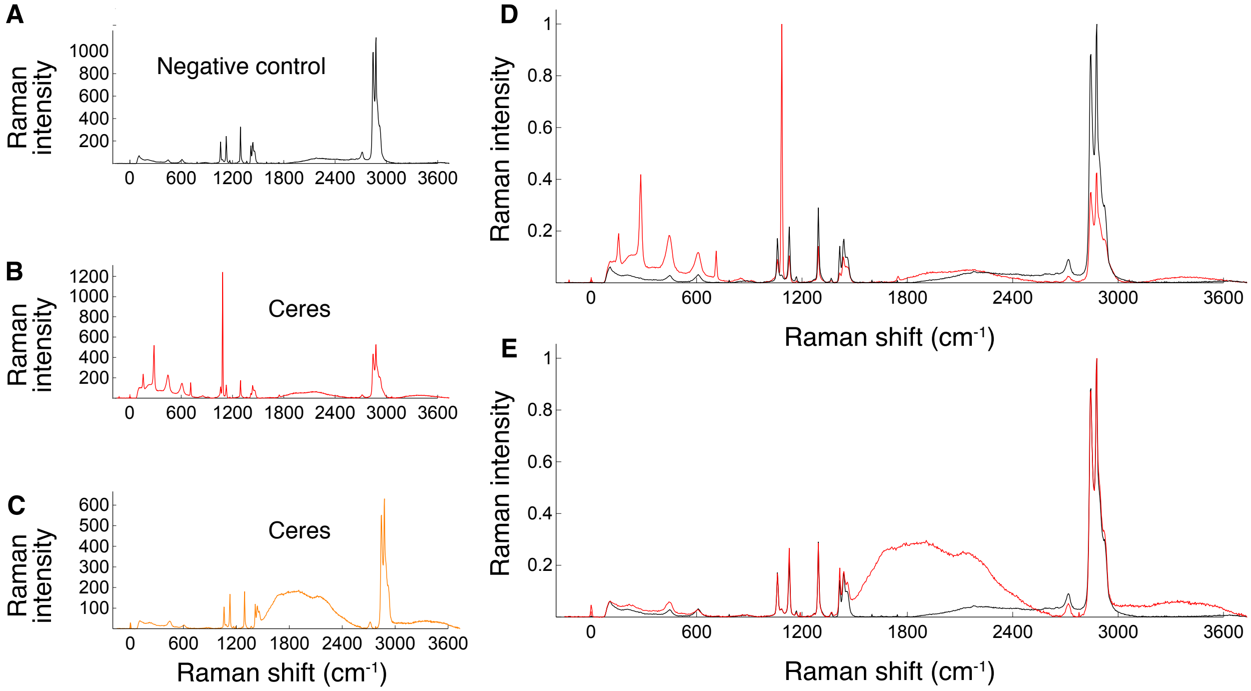

Supplementary Fig. 6. Ceres effect on PE film. A, B AFM-RAMAN spectroscopy of control (A) and Ceres treated PE film (B, C). A. Control PE film, showing the typical PE peaks at 1061, 1128, 1294, 1440, 2846 and 2880 cm^{-1}. B, C. Punctual analyses of treated PE film, indicating PE deterioration. The spectrum shows an intense peak at 1085 cm^{-1} assigned to amorphous PE (B). The peaks at 711 and 1747 cm^{-1} correspond to the C=O group. D, E. Overlapping of the negative control with the experimental sample in B (D), and C (E). The broad band between 1500 and 2400 cm^{-1} (B, C) indicates different collective stretching vibrations due to the presence of other organic compounds, that is PE deterioration. Source data are provided as a Source Data file.

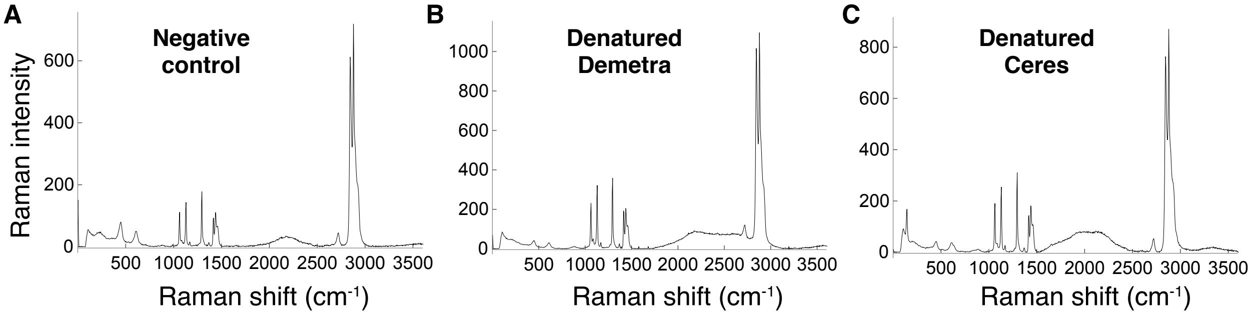

Supplementary Fig. 7. Denatured Demetra and Ceres on PE film. A-C. RAMAN spectroscopy of control PE (A) and PE treated with denatured Demetra (B) and Ceres (C). Source data are provided as a Source Data file.

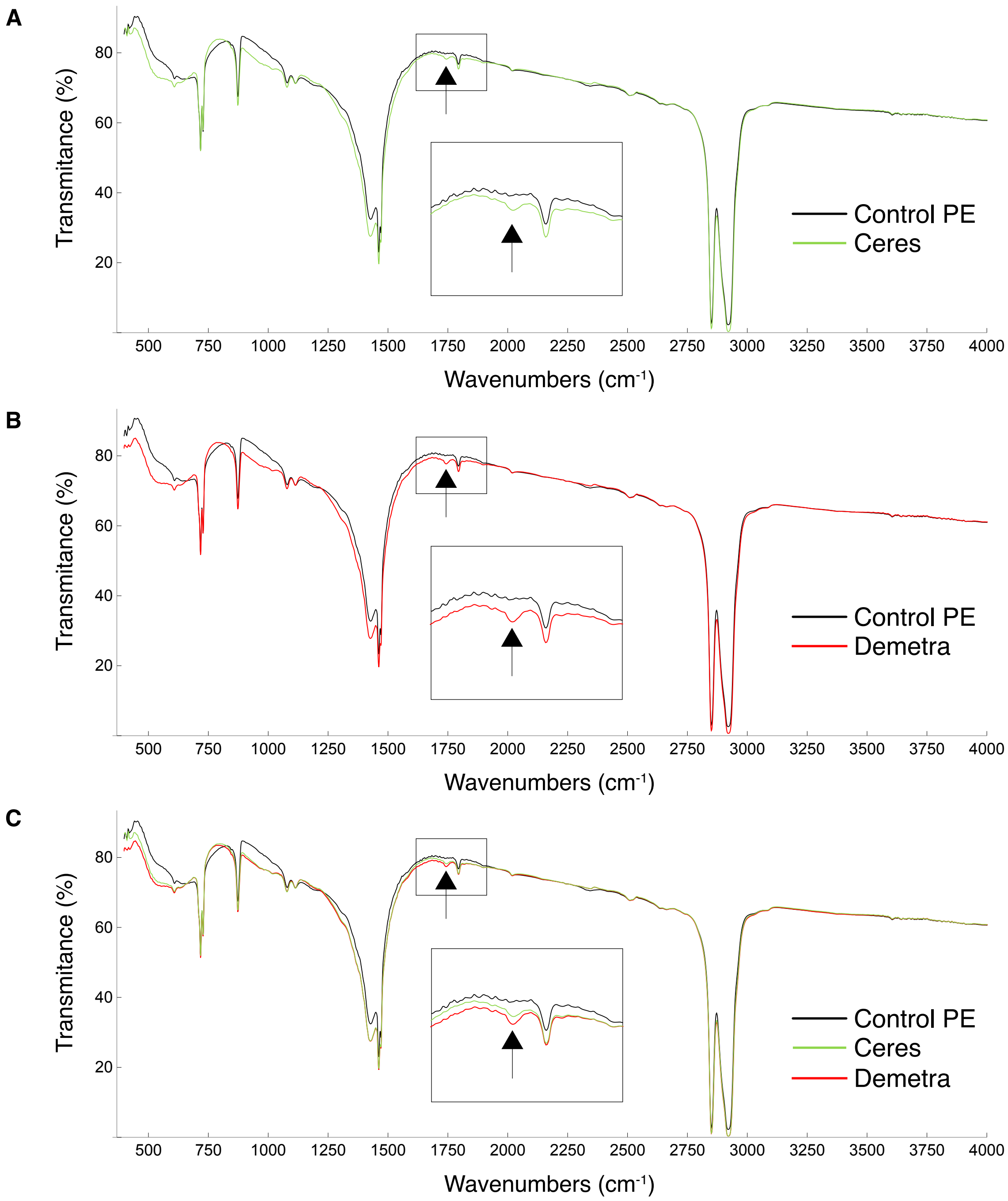

Supplementary Fig. 8. FTIR of PE film treated with Demetra and Ceres. A. Ceres and control PE. B. Demetra and control PE. C. The two PEases and control PE. The arrow indicates the peak

at around 1750 cm^{-1} (i.e. oxidation-carbonyl group). Source data are provided as a Source Data file.

A

Name	Start	End	E-value
Pfam:Hemocyanin_N	33	155	1.8e-40
Pfam:Hemocyanin_M	160	438	2.7e-73
Pfam:Hemocyanin_C	447	679	1.5e-69

B

Name	Start	End	E-value
Signal peptide	1	18	N/A
Pfam:Hemocyanin_N	32	155	2.3e-30
Pfam:Hemocyanin_M	160	437	6.4e-78
Pfam:Hemocyanin_C	446	692	8.9e-63

C

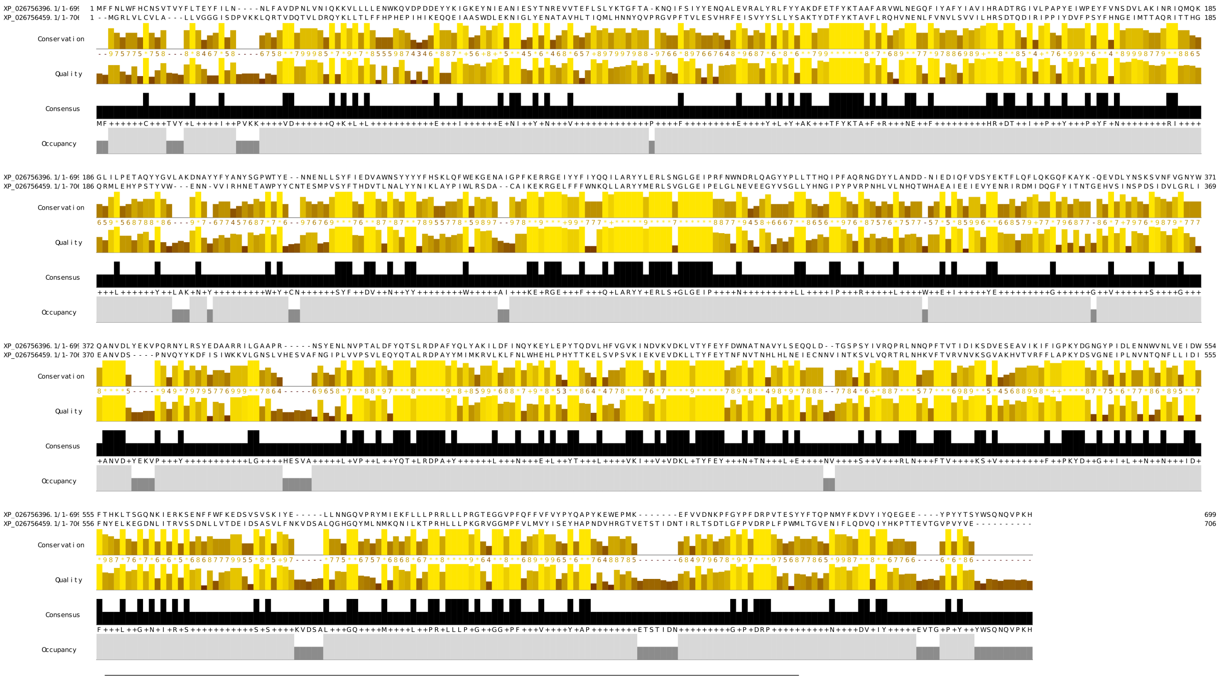

D

Feature	Demetra	Ceres
Length (aa)	699	706
MW	83654.31	81581.47
pI	5.12	6.13
Total number of negatively charged residues (Asp + Glu)	86	76
Total number of positively charged residues (Arg + Lys)	68	62
Estimated half-life (mammalian reticulocytes, in vitro)	30 hours	30 hours
Instability Index	35.98 (stable)	41.69 (unstable)
Aliphatic index	80.03	100.78

Supplementary Fig. 9. Comparison between Demetra and Ceres. A, B. Prediction of functional domains of Demetra (A) and Ceres(B) with SMART.C. Pairwise alignment of Ceres and Demetra with Clustal Omega. D. Comparison of general statistics obtained from Expasy ProtParam.

Supplementary Table 1. Molecular weight (uma), fragment ions (m/z), and intensity of the untargeted detected compounds from the NIST Mass Spectrometry library compared to those detected in our study.

Molecule	Molecular weight (uma)	Mass spectrum (NIST Mass Spectrometry library) m/z (intensity)					Mass spectrum of the sample m/z (intensity)				
**2-decanone (10 C)**	156	58 (99.9)	43 (84.5)	71 (37.7)	59 (27.9)	41 (27.3)	58 (99.9)	43 (92.0)	41 (51.2)	44 (44.7)	55 (44.3)
**2-dodecanone (12 C)**	184	58 (99.9)	43 (93.4)	71 (38.7)	59 (34.4)	41 (32.3)	58 (99.9)	43 (46.1)	59 (34.3)	41 (22.7)	71 (22.1)
**2-tetradecanone (14 C)**	212	58 (99.9)	43 (96.3)	59 (43.1)	71 (34.6)	41 (31.6)	58 (99.9)	43 (61.6)	59 (38.3)	71 (36.5)	41 (26.6)
**2-hexadecanone (16 C)**	240	58 (99.9)	59 (61.9)	43 (59.4)	71 (39.3)	41 (25.8)	58 (99.9)	43 (86.8)	59 (59.7)	71 (41.2)	41 (30.4)
**2-octadecanone (18 C)**	268	58 (99.9)	59 (75.2)	43 (59.7)	71 (30.7)	41 (23.7)	43 (99.9)	58 (87.7)	71 (65.8)	59 (54.4)	41 (51.3)
**2-eicosanone (20 C)**	296	-	-	-	-	-	58 (99.9)	43 (82.7)	71 (73.4)	41 (69.3)	55 (64.6)
**2-docosanone (22 C)**	324	-	-	-	-	-	58 (99.9)	59 (93.8)	43 (75.9)	57 (67.8)	44 (6.22)
**2,3-Butanediol, 2TMS derivative**	234	117 (99.9)	73 (83.3)	147 (34.2)	75 (15.1)	118 (11.0)	117 (99.9)	73 (59,2)	147 (37.5)	75 (9.4)	118 (9.0)
**Benzenepropanoic acid,TMS derivative**	222	104 (99.9)	75 (85.0)	73 (47.0)	207 (41.5)	91 (27.0)	104 (99.9)	75 (80.2)	73 (47.8)	207 (36.1)	91 (30.0)
**Sebacic acid, 2TMS derivative**	346	331 (99.9)	73 (87.4)	75 (78.0)	215 (49.7)	129 (38.1)	331 (99.9)	215 (78.2)	73 (67.4)	75 (40.3)	129 (16.5)

Supplementary Table 2. BLAST search of the NCBI proteins that show some sequence identity with Demetra and Ceres and whose structure has been characterized in the literature.

	Entity ID	Protein	DOI	Link	Sequence Alignment
Ceres	4L37_2	Storage protein *(Bombyx mori)*	10.1107/S0907444913021823	4L37	Seq. Identity: 30%, E-Value: 2.35e-87, Region: 12-664
Ceres	3WJM_1	Arylphorin *(B. mori)*	10.1002/pro.2457	3WJM	Seq. Identity: 30%, E-Value: 5.119e-88, Region: 6-680
Ceres	3GWJ_1	Arylphorin *(Antheraea pernyi)*	10.1042/BJ20082170	3GWJ	Seq. Identity: 30%, E-Value: 4.161e-85, Region: 6-658
Demetra	4L37_1	Storage protein *(B. mori)*	10.1107/S0907444913021823	4L37	Seq. Identity: 54%, E-Value: 5.652e-243, Region: 11-666
Demetra	4L37_2	Storage protein *(B. mori)*	10.1107/S0907444913021823	4L37	Seq. Identity: 57%, E-Value: 3.035e-246, Region: 14-686
Demetra	3WJM_1	Arylphorin *(B. mori)*	10.1002/pro.2457	3WJM	Seq. Identity: 57%, E-Value: 3.035e-246, Region: 30-702
Demetra	3WJM_2	Arylphorin *(B. mori)*	10.1002/pro.2457	3WJM	Seq. Identity: 54%, E-Value: 5.652e-243, Region: 27-682
Demetra	3GWJ_1	Arylphorin *(A. pernyi)*	10.1042/BJ20082170	3GWJ	Seq. Identity: 57%, E-Value: 2.453e-244, Region: 8-664

Supplementary Data Legends

Supplementary Data 1. Protein content of whole ww saliva.

Supplementary Data 2. Proteins in the wax worm saliva. Protein content of ion exchange (peak 1, 2c and 3), and size exclusion (peak 5) chromatographic column peaks. The protein content of whole saliva is included in the incorporated file (icon above)

Supplementary Data 3. BLAST search of the NCBI proteins with sequence identity (> 50%) with Demetra.

Supplementary Data 4. BLAST search of the NCBI proteins with sequence identity (> 50%) with Ceres.
