# Supplementary material for: Wax worm saliva and the enzymes therein are the key to polyethylene degradation by Galleria mellonella
Source: Nat Commun. 2022 Oct 4;13:5568. doi: 10.1038/s41467-022-33127-w (PMC9532405; doi:10.1038/s41467-022-33127-w)
Supplement: Supplementary file 3 — Description of additional Supplementary File [file 41467_2022_33127_MOESM3_ESM.pdf]

Descriptions of Additional Supplementary Data Files

Supplementary Data 1. Protein content of whole ww saliva.

Supplementary Data 2. Proteins in the wax worm saliva. Protein content of ion exchange (peak 1, 2c and 3), and size exclusion (peak 5) chromatographic column peaks. The protein content of whole saliva is included in the incorporated file (icon above)

Supplementary Data 3. BLAST search of the NCBI proteins with sequence identity (> 50%) with Demetra.

Supplementary Data 4. BLAST search of the NCBI proteins with sequence identity (> 50%) with Ceres.
