# Supplementary material for: Wax worm saliva and the enzymes therein are the key to polyethylene degradation by Galleria mellonella
Source: Nat Commun. 2022 Oct 4;13:5568. doi: 10.1038/s41467-022-33127-w (PMC9532405; doi:10.1038/s41467-022-33127-w)
Supplement: Supplementary file 8 — Reporting Summary [file 41467_2022_33127_MOESM8_ESM.pdf]

nature portfolio

Corresponding author(s): Federica Bertocchini

Last updated by author(s): Aug 22, 2022

Reporting Summary

Nature Portfolio wishes to improve the reproducibility of the work that we publish. This form provides structure for consistency and transparency in reporting. For further information on Nature Portfolio policies, see our Editorial Policies and the Editorial Policy Checklist.

Statistics

For all statistical analyses, confirm that the following items are present in the figure legend, table legend, main text, or Methods section.

n/a	Confirmed	
☒	☐	The exact sample size (*n*) for each experimental group/condition, given as a discrete number and unit of measurement
☒	☐	A statement on whether measurements were taken from distinct samples or whether the same sample was measured repeatedly
☒	☐	The statistical test(s) used AND whether they are one- or two-sided<br>*Only common tests should be described solely by name; describe more complex techniques in the Methods section.*
☒	☐	A description of all covariates tested
☒	☐	A description of any assumptions or corrections, such as tests of normality and adjustment for multiple comparisons
☒	☐	A full description of the statistical parameters including central tendency (e.g. means) or other basic estimates (e.g. regression coefficient) AND variation (e.g. standard deviation) or associated estimates of uncertainty (e.g. confidence intervals)
☒	☐	For null hypothesis testing, the test statistic (e.g. *F*, *t*, *r*) with confidence intervals, effect sizes, degrees of freedom and *P* value noted<br>*Give P values as exact values whenever suitable.*
☒	☐	For Bayesian analysis, information on the choice of priors and Markov chain Monte Carlo settings
☒	☐	For hierarchical and complex designs, identification of the appropriate level for tests and full reporting of outcomes
☒	☐	Estimates of effect sizes (e.g. Cohen's *d*, Pearson's *r*), indicating how they were calculated

Our web collection on statistics for biologists contains articles on many of the points above.

Software and code

Policy information about availability of computer code

Data collection	
Data analysis	The chromatograms were processed using the software MSD ChemStation E.01.00.237 from Agilent Technologies, Inc.<br>Proteome Discoverer (version 1.4.1.14) was used for protemics mass spectrometry.<br>The RNA-seq data was mapped on the reference genome using STAR (version 2.5 0c) in local mode and used to perform a reference guided transcriptome assembly with Trinity (v2.11.0).<br>Protein sequences were aligned using Clustal Omega and then visualized with Jalview.<br>The prediction of the biochemical characteristics of the proteins was performed with Expasy's ProtParam tool.

For manuscripts utilizing custom algorithms or software that are central to the research but not yet described in published literature, software must be made available to editors and reviewers. We strongly encourage code deposition in a community repository (e.g. GitHub). See the Nature Portfolio guidelines for submitting code & software for further information.

Data

Policy information about availability of data

All manuscripts must include a data availability statement. This statement should provide the following information, where applicable:

- Accession codes, unique identifiers, or web links for publicly available datasets
- A description of any restrictions on data availability
- For clinical datasets or third party data, please ensure that the statement adheres to our policy

The data generated or analysed during this study are included in this published article (and its supplementary information files). In addition, the RNA-seq data that was used for the G. mellonella genome annotation have been deposited in NCBI under the BioProject accession number PRJNA822887. Also, the newly generated annotation in GFF3/GTF format was deposited in Data Mendeley (doi: 10.17632/t7b5s58vxt.2)

Human research participants

Policy information about studies involving human research participants and Sex and Gender in Research.

Reporting on sex and gender	n/a
Population characteristics	n/a
Recruitment	n/a
Ethics oversight	n/a

Note that full information on the approval of the study protocol must also be provided in the manuscript.

Field-specific reporting

Please select the one below that is the best fit for your research. If you are not sure, read the appropriate sections before making your selection.

[x] Life sciences [ ] Behavioural & social sciences [ ] Ecological, evolutionary & environmental sciences

For a reference copy of the document with all sections, see nature.com/documents/nr-reporting-summary-flat.pdf

Life sciences study design

All studies must disclose on these points even when the disclosure is negative.

Sample size	The experiments were qualitative. Experiments were repeated several times, as indicated in the manuscript. The results were consistent in all the repetitions
Data exclusions	No data have been excluded
Replication	All attempts of replication were successful
Randomization	n/a
Blinding	Blinding design was incorporated in all the analyses

Reporting for specific materials, systems and methods

We require information from authors about some types of materials, experimental systems and methods used in many studies. Here, indicate whether each material, system or method listed is relevant to your study. If you are not sure if a list item applies to your research, read the appropriate section before selecting a response.

Materials & experimental systems

n/a	Involved in the study
☒	☐ Antibodies
☒	☐ Eukaryotic cell lines
☒	☐ Palaeontology and archaeology
☐	☒ Animals and other organisms
☒	☐ Clinical data
☒	☐ Dual use research of concern

Methods

n/a	Involved in the study
☒	☐ ChIP-seq
☒	☐ Flow cytometry
☒	☐ MRI-based neuroimaging

Animals and other research organisms

Policy information about studies involving animals; ARRIVE guidelines recommended for reporting animal research, and Sex and Gender in Research

Laboratory animals	The experiments were performed using saliva of insect lepidopter Galleria mellonella, raised in the laboratory as described.
Wild animals	n/a
Reporting on sex	n/a
Field-collected samples	n/a
Ethics oversight	Insect larvae are not subject to ethical regulation

Note that full information on the approval of the study protocol must also be provided in the manuscript.
